# A Broadly Reactive One-Step SYBR Green I Real-Time RT-PCR Assay for Rapid Detection of Murine Norovirus

**DOI:** 10.1371/journal.pone.0098108

**Published:** 2014-05-21

**Authors:** Ken-Ichi Hanaki, Fumio Ike, Ayako Kajita, Wataru Yasuno, Misato Yanagiba, Motoki Goto, Kouji Sakai, Yasushi Ami, Shigeru Kyuwa

**Affiliations:** 1 Department of Laboratory Animal Medicine, Institute for Biomedical Sciences, Iwate Medical University, Yahaba, Iwate, Japan; 2 Center for In Vivo Sciences, Institute for Biomedical Sciences, Iwate Medical University, Yahaba, Iwate, Japan; 3 Experimental Animal Division, RIKEN BioResource Center, Tsukuba, Ibaraki, Japan; 4 Center for Disease Biology and Integrative Medicine, Faculty of Medicine, The University of Tokyo, Bunkyo, Tokyo, Japan; 5 Department of Virology III, National Institute of Infectious Diseases (NIID), Musashimurayama, Tokyo, Japan; 6 Division of Experimental Animal Research, NIID, Shinjuku, Tokyo, Japan; 7 Department of Biomedical Science, Graduate School of Agricultural and Life Sciences, The University of Tokyo, Bunkyo, Tokyo, Japan; University of Malaya, Malaysia

## Abstract

A one-step SYBR Green I real-time RT-PCR assay was developed for the detection and quantification of a broad range of murine noroviruses (MNVs). The primer design was based on the multiple sequence alignments of 101 sequences of the open reading frame (ORF)1−ORF2 junction of MNV. The broad reactivity and quantitative capacity of the assay were validated using 7 MNV plasmids. The assay was completed within 1 h, and the reliable detection limit was 10 copies of MNV plasmid or 0.063 median tissue culture infective doses per milliliter of RAW264 cell culture-propagated viruses. The diagnostic performance of the assay was evaluated using 158 mouse fecal samples, 91 of which were confirmed to be positive. The melting curve analysis demonstrated the diversity of MNV in the samples. This is the first report of a broadly reactive one-step SYBR Green I real-time RT-PCR assay for detecting of MNVs. The rapid and sensitive performance of this assay makes it a powerful tool for diagnostic applications.

## Introduction

Murine norovirus (MNV), a non-enveloped virus with a positive-sense single-stranded RNA genome, is a member of the genus *Norovirus* in the *Caliciviridae* family. MNV-1 was first discovered as a cause of lethality in severely immunocompromised mice that lacked recombination-activating gene 2 and signal transducer and activator of transcription 1 (STAT1) [1]. Thereafter, many MNV strains have been detected and/or isolated from laboratory mice and wild rodents [2]–[6]. Today, MNV is recognized as the most prevalent infectious agent in laboratory mouse colonies worldwide [7]–[12]. MNV shows considerable genetic diversity, which influences infectivity and virulence [13]–[16]. For example, MNV-1 causes a lethal infection with symptoms such as encephalitis, hepatitis, and pneumonia in STAT1 knockout mice, whereas MNV-O7 causes a subclinical infection in these mice [17]. MNV-1 causes a transient infection without symptoms [1], [4], [8] or with dose-dependent mild diarrhea in immunocompetent mice [18], whereas most MNV strains cause a long-term asymptomatic infection in immunocompetent mice with fecal viral shedding [4], [16]. However, MNV infection does not alter the intestinal microbiota of immunocompetent mice [19].

Whether MNV infection interferes with the results of research is of great concern to biomedical researchers. Thus, the effects of MNV infection on biomedical research have been investigated. MNV-4 infection was observed to accelerate the progression of bacteria-induced inflammatory bowel disease in the multidrug resistance gene *mdr1a* knockout mice but it did not modulate the progression of inflammatory bowel disease in the Smad3 knockout mice [20]. MNV-4 but not MNV-CW3 infection induced multiple inflammatory hallmarks of human Crohn's disease in Atg16L1^HM^ mice after dextran sodium sulfate administration [21]. A model of intestinal inflammation and fibrosis induced by *Salmonella* Typhimurium infection in C57BL/6 mice was not dramatically altered by either MNV-1 or MNV-4 co-infection [22]. MNV-CR6 did not alter immune responses in C57BL/6 mice co-infected with Friend virus [23], influenza A virus [24], vaccinia virus [24] or murine cytomegalovirus [25]. As these previous studies indicate that the effects of MNV infection on experiments in mice cannot be generalized, MNV-free mice are recommended for use in biomedical research. Thus, MNV infection in laboratory mouse colonies are monitored periodically by immunological methods such as the enzyme-linked immunosorbent assay [9] and multiplexed fluorescent immunoassay [8], because MNV is believed to comprise a single serogroup [16]. MNV infection in colonies may also be monitored by conventional reverse transcription-polymerase chain reaction (RT-PCR) assays including nested RT-PCR [8], [26]–[30]. However, conventional RT-PCR assays are time consuming, laborious, inconvenient, and prone to false positives due to cross-contamination. In addition, RT-PCR assays need to be updated for detecting all currently known MNV isolates due to their considerable genetic diversity [2], [11], [16].

MNV, especially MNV-1, is often used as a substitute for human norovirus (HuNoV) because of the absence of a cell culture system and animal infection model [31]–[34]. MNV can be cultivated easily in cell cultures [35], and infectious titers can be quantified by plaque assays and median tissue culture infective dose (TCID_50_) [9], [36], [37]. MNV is similar to HuNoV with respect to its morphology, genetics, and replication cycle [31], [35]. Thus, real-time RT-PCR assays have been developed for the detection and quantification of MNV-1 RNA [31], [32], [38]–[40]. However, these quantitative assays are not applicable for detecting MNV infection in laboratory mouse colonies because of the genetic diversity of MNV. A broadly reactive two-step TaqMan real-time RT-PCR assay (TaqMan assay) has been developed for the detection of numerous MNV strains [41]. However, the two-step assay is not suitable for routine monitoring of MNV infection in laboratory mouse colonies because it is time-consuming, laborious, and allows for increased opportunities of DNA contamination. Although the primers and probe were designed on the basis of the multiple nucleotide sequence alignments of 44 MNV nucleotide sequences from the highly conserved ORF1−ORF2 junction, this region still contains a number of polymorphisms among all currently known MNV isolates that may interfere in accurate amplification and quantification of the assay.

The objective of this study was to develop a one-step SYBR Green I real-time RT-PCR assay (SYBR Green I assay) for the detection of all currently known MNV strains. To achieve this, a set of primers was designed based on the multiple sequence alignments of the available 101 nucleotide sequences of the ORF1−ORF2 junction of MNV. The diagnostic performance of this assay was compared with that of the TaqMan assay in the detection of MNV in 158 mouse fecal samples derived from 25 different laboratories or institutes in Japan, the United States, and 3 European countries. The results indicate that the SYBR Green I assay is broadly reactive, rapid, sensitive, and accurate in the laboratory diagnosis of MNV infection.

## Materials and Methods

### Ethics statement

The mouse fecal samples used in this study were collected from 158 mouse cages (each housing 1–4 mice) housed in reproduction rooms for rederivation at RIKEN BioResource Center (RIKEN BRC), one of the founding members of the Federation of International Mouse Resources (FIMRe). A total of 76 mouse strains (58 strains of C57BL/6 or its mixed backgrounds and 18 strains with other inbred or closed colony backgrounds) were deposited at RIKEN BRC from 19 laboratories or institutes in Japan, 2 laboratories each in the United States and France, and 1 laboratory each in Germany and Denmark. The collection of fecal samples was conducted according to the RIKEN guidelines for animal research and was approved by the Animal Experimentation Committee at the RIKEN Tsukuba Institute (Approval no. JITSU13-002).

### Viruses and RAW264 cells

MNV-1.CW1 (1.CW1) [42], obtained from the American Type Culture Collection, and MNV-S7-PP3 (S7-PP3) [9] were used in this study. RAW264 cells were maintained in Dulbecco's Modified Eagle's Medium (DMEM) supplemented with 10% (v/v) fetal bovine serum, 2 mM L-glutamine, and 50 µg/mL gentamicin at 37°C in a humidified 5% CO_2_ atmosphere. The viruses were grown in RAW264 cells and titrated as described previously [43].

### Preparation of viral RNA from cell culture supernatants and mouse fecal samples

To prepare MNV RNA standards, 10-fold serially diluted virus stocks of 1.CW1 and S7-PP3 were prepared with serum-free DMEM in triplicates, respectively. To extract RNA from mouse fecal samples, fecal supernatants were obtained by centrifugation of 6.5–320 mg/mL fecal suspensions in PBS at 5000 ×*g* for 5 min. MNV RNA was extracted from 200 ìL of each supernatant using a High Pure Viral RNA Kit (Roche Applied Science, Minato, Tokyo, Japan) according to the manufacturer's instructions. Control viral RNA was also prepared from 2×10^6^ plaque forming units/mL of diarrhea virus of infant mice (DVIM) culture supernatants and 1.3×10^7^ TCID_50_/mL of feline calicivirus (FCV) culture supernatants using the kit.

### Construction of standard plasmids

A 400-bp DNA fragment of the ORF1–ORF2 junction of 7 MNV strains was chemically synthesized, cloned into a pUC19 vector, and confirmed by DNA sequencing. The strains and nucleotide sequence positions used in the construction were as follows: nt 4801–5200 of 1.CW1 (GenBank accession no. DQ285629), Berlin/04/06 (DQ911368), KHU-1 (JX048594), and S7-PP3 (AB435515); nt 4527–4926 of TW2006 (EU482057) and TW2007 (EU482058); and nt 4776–5175 of Apo960 (JN975492). They were manufactured by Fasmac (Atsugi, Kanagawa, Japan). To construct MNV plasmid standards, each plasmid was 10-fold serially diluted at final concentrations of 1.0×10^8^–10 copies/5 µL of the elution buffer from the viral RNA purification kit.

### Primer design

A total of 101 sequences from the ORF1–ORF2 junction of MNV were obtained from GenBank and aligned with Genetyx-Mac v16 (Genetyx Corp., Shibuya, Tokyo, Japan). Regions of the genomes that were highly conserved among all the viruses were used to design primers. The forward and reverse primer sequences are F2 (5′-ATGGTRGTCCCACGCCAC-3′) and R2 (5′-TGCGCCATCACTCATCC-3′), respectively, with R representing a purine (A or G). All the primers used in this study were synthesized by Life Technologies (Chuo, Tokyo, Japan).

### SYBR Green I assay

The SYBR Green I assay was performed using a One Step SYBR PrimeScript PLUS RT-PCR Kit (Takara BIO, Otsu, Shiga, Japan) and was carried out in a 20 µL reaction which consisted of 5 µL viral RNA or MNV plasmid DNA, 0.5 µM each of F2 and R2 primers, 1.2 µL TaKaRa Ex Taq HS Mix, 0.4 µL PrimeScript PLUS RTase Mix in 1× One Step SYBR RT-PCR Buffer 4. For the negative controls, viral RNA was substituted with FCV or DVIM RNA, and MNV plasmid DNA was substituted with pUC19 DNA or molecular grade water. The SYBR Green I assay was performed using a LightCycler Nano (Roche Applied Science, Minato, Tokyo, Japan) with an initial incubation at 42°C for 5 min for RT followed by denaturation at 95°C for 10 s. Forty cycles of amplification were performed using a thermal cycling profile of 94°C for 10 s, 57°C for 10 s, and 72°C for 30 s. Subsequently, a melting curve was recorded by holding at 95°C for 60 s, cooling to 60°C for 20 s, and then heating at 0.1°C/s up to 95°C. The amplification and melting curve data were collected and analyzed using the LightCycler Nano software 1.0.

### TaqMan assay

The TaqMan assay developed by Kitajima et al. [41] was performed with modifications. The RT reaction was performed using a High Capacity cDNA Reverse Transcription Kit (Life Technologies) according to the manufacturer's instructions. One-tenth of the RT product (20 µL) was used in the real-time PCR with Premix Ex Taq for probe qPCR (Takara Bio) using 0.4 µM of each of the primers (forward, 5′-CCGCAGGAACGCTCAGCAG-3′; reverse, 5′-GGYTGAATGGGGACGGCCTG-3′) and 0.3 µM TaqMan MGB probe (5′-FAM-ATGAGTGATGGCGCA-MGB/NFQ-3′, Life Technologies). PCR amplification was performed using the LightCycler Nano system: initial denaturation at 95°C for 30 s to activate DNA polymerase, followed by 40 cycles of amplification with denaturation at 95°C for 15 s, and annealing and extension at 60°C for 60 s.

## Results

### Selection of a primer pair

**Figure 1:**
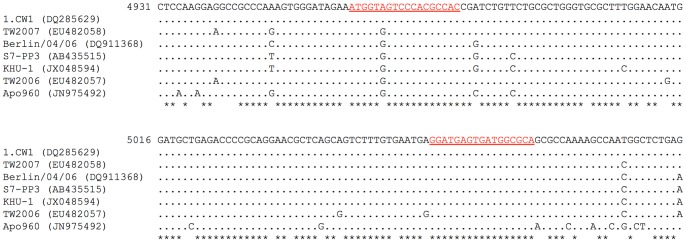
Alignment of nucleotide sequences from the ORF1–ORF2 junctions of 7 MNV. Nucleotide sequences of 7(nt 4962–4979) and R2 (nt 5060–5076) are indicated in the underlined red text.

Nucleotide sequences of the ORF1–ORF2 junction of 101 MNV, including the sequences of 8 MNV-like viruses detected by RT-PCR in wild rodents in the United Kingdom and Japan, were aligned to identify highly conserved regions. The strains and their GenBank accession numbers are shown in Table S1. The forward and reverse primers were then designed manually on the basis of the following 4 criteria: (i) primers of 17–20 bases in length; (ii) the last 10 nucleotides at the 3′ end of primers completely matched the aligned sequence; (iii) the last nucleotide of the 3′ end of primers is C or G; and (iv) the PCR product is 60–220 bp in length. A total of 11 sets of primer pairs were initially examined for the success of amplification in the SYBR Green I assay using the S7-PP3 RNA extracted from cell culture supernatants. The absence of non-specific products and primer dimers was confirmed by the melting curve analysis. Finally, a set containing primer pair F2 and R2 that amplified a 115-bp fragment of the ORF1–ORF2 junction was selected for the greatest sensitivity and specificity (data not shown). The primer locations are shown in Figure 1.

### Sensitivity, dynamic range, and linearity

The performance of the SYBR Green I assay was determined using 7 different 10-fold serially diluted MNV plasmids from 1×10^8^ copies to 10 copies per reaction. Each dilution was subjected to the assay in triplicates, and standard curves were generated by plotting the threshold cycle (Ct) values against the different copy numbers of plasmid DNA. The quantitative amplification parameters for the respective assays are shown in Table 1. In summary, the assay had a 7-log linear dynamic range (r^2^≥0.992) and a detection limit of at least 10 copies of plasmid DNA. The amplification efficiency ranging between 101% and 111%, which corresponds to a slope between −3.29 and −3.09, was acceptable for the MNV gene quantification.

**Table 1:** Standard curves of SYBR Green I assay using 7 different plasmids.

	Amplification parameter				
Plasmid	Slope	Y-intercept	R square	Efficiency (%)	Linear range (no. of copies)
1.CW1	−3.19	37.93	0.997	106	10–10^8^
Apo960	−3.14	38.54	0.996	108	10–10^8^
Berlin/04/06	−3.25	38.77	0.996	103	10–10^8^
KHU-1	−3.10	38.46	0.992	110	10–10^8^
S7-PP3	−3.29	39.22	0.997	101	10–10^8^
TW2006	−3.09	39.47	0.994	111	10–10^8^
TW2007	−3.18	38.82	0.997	106	10–10^8^

### Accurate quantification and melting curve analysis

**Figure 2:**
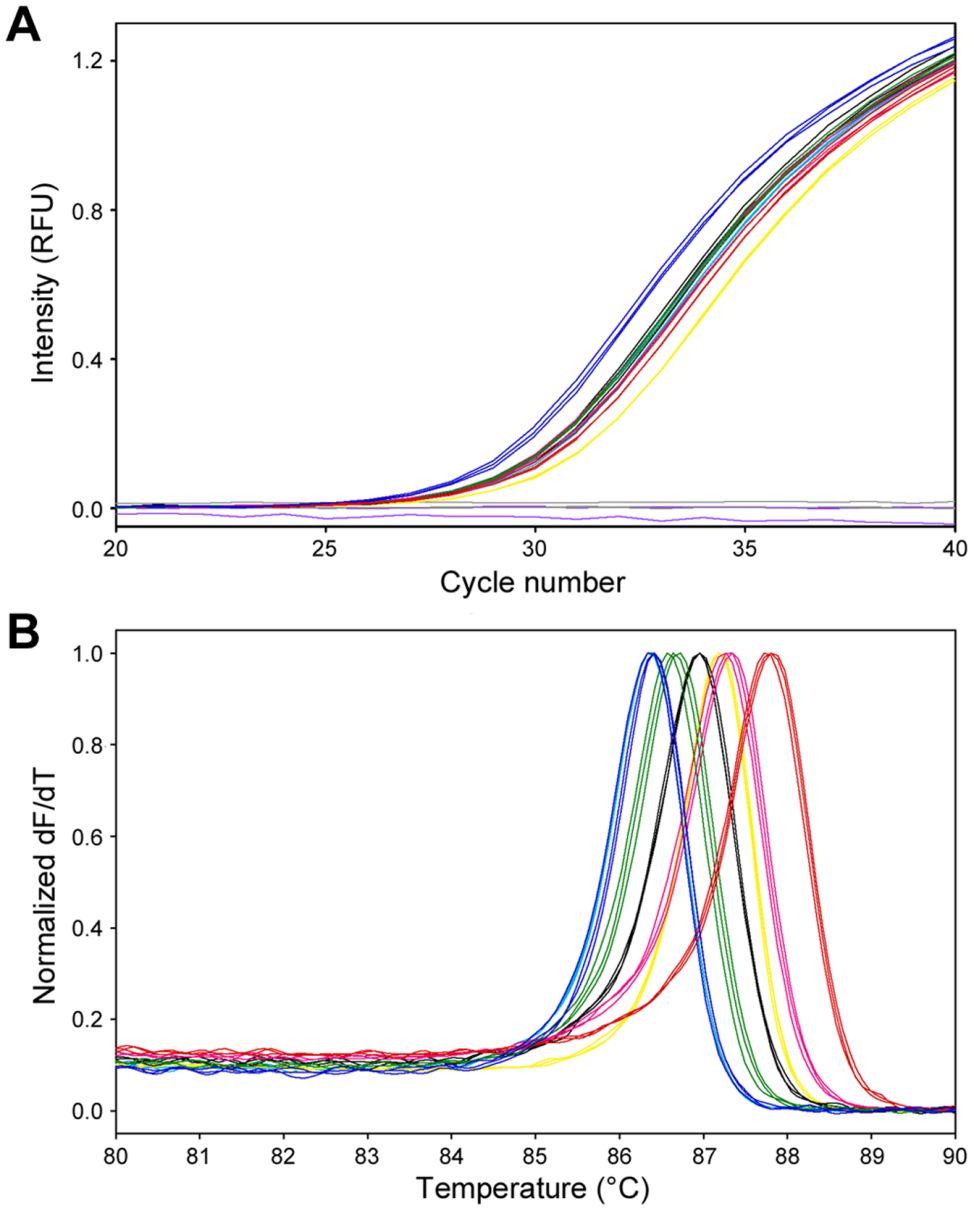
SYBR Green I assay followed by melting curve analysis of 7 (A) Amplification kinetics results with 1000 copies of the 7 MNV plasmids, and (B) melting curve analysis results of 7 MNV PCR products. 1.CW1, blue; Apo960, red; Berlin/04/06, green; KHU-1, magenta; S7-PP3, black; TW2006, yellow; TW2007, cyan. As negative controls, pUC19 DNA (purple) and molecular grade water (gray) are shown in A. All samples were tested in triplicates.

For the melting curve analysis, SYBR Green I assays were performed using 1000 copies of the 7 different MNV plasmids per reaction in triplicates. Figure 2A shows that amplification efficiencies were comparable between the 7 MNV plasmids (mean Ct values ± standard deviation: 28.09±0.10 for 1.CW1, 29.21±0.09 for Apo960, 28.85±0.03 for Berlin/04/06, 28.87±0.10 for KHU-1, 28.86±0.10 for S7-PP3, 29.75±0.01 for TW2006, and 29.01±0.11 for TW2007). The non-specific gene amplification was not observed when MNV plasmid DNA was substituted with 1×10^11^ copies of pUC19 DNA or molecular grade water. However, Figure 2B shows apparent similarities and differences in melting temperature (T_m_) values between the 7 MNV plasmids (mean T_m_ values ± standard deviation: 86.37±0.06°C for 1.CW1, 87.77±0.07°C for Apo960, 86.65±0.05°C for Berlin/04/06, 87.30±0.04°C for KHU-1, 86.95±0.01°C for S7-PP3, 87.19±0.01°C for TW2006, and 86.37±0.01°C for TW2007). Of 101 MNV strains, PCR products of 1.CW1, TW2007, and 42 other strains were expected to show the lowest T_m_ values, and the product of Apo960 was expected to show the highest T_m_ values. The T_m_ values obtained were in agreement with those of the respective nucleotide composition of PCR products, excluding primer regions (Figure 1).

### Correlation between virus titer and Ct value

**Figure 3:**
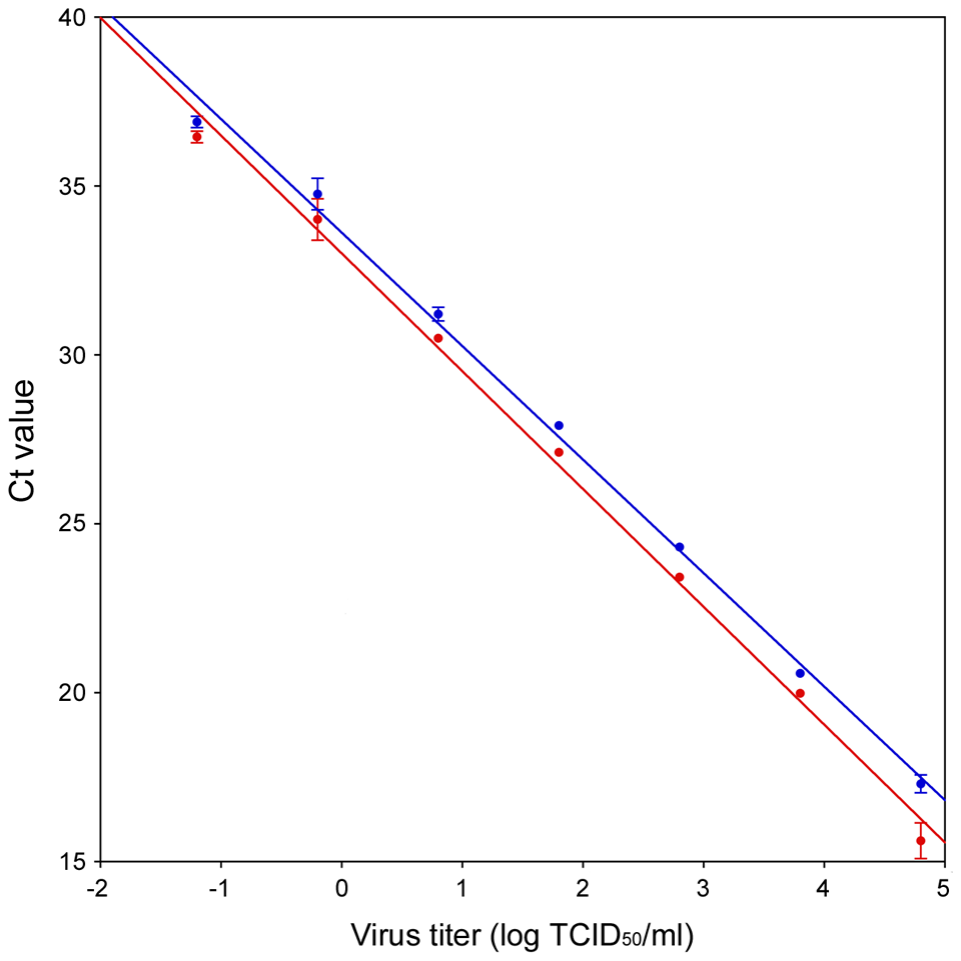
Relationship between virus titer and Ct value. Viral RNA was extracted from triplicate 10-fold serially diluted virus stocks from 6.32×10^4^ to 6.32×10^−3^ TCID_50_/mL of 1.CW1 and S7-PP3, respectively. Data are presented as log TCID_50_ and the mean of three samples for Ct values. 1.CW1, blue; S7-PP3, red; y-error bars, standard deviations.

The relationship between MNV infectious titers and Ct values was also examined. Viral RNA samples were extracted from 10-fold serially diluted cell culture supernatants of 1.CW1 and S7-PP3 in triplicates, respectively, and were used to create standard curves generated by the SYBR Green I assay for viral infectious titers. The results showed that the SYBR Green I assay had a 6-log linear dynamic range between TCID_50_ titers and Ct values (Figure 3). The standard curve obtained for 1.CW1 showed an amplification efficiency of 93.4%, a regression coefficient of 0.996, a slope of −3.49, and an intercept of 33. The standard curve obtained for S7-PP3 showed an amplification efficiency of 98.4%, a regression coefficient of 0.997, a slope of −3.36 and an intercept of 33.61. The T_m_ values were 86.30±0.09°C for 1.CW1 and 86.83±0.07°C for S7-PP3. The detection limits of 1.CW1 and S7-PP3 by the SYBR Green I assay were 6.32×10^−2^ TCID_50_/mL (1.26×10^−3^ TCID_50_/reaction), estimated as 3 and 5 copies, respectively, using corresponding MNV plasmid standards.

### Detection of MNV in mouse fecal samples

**Figure 4:**
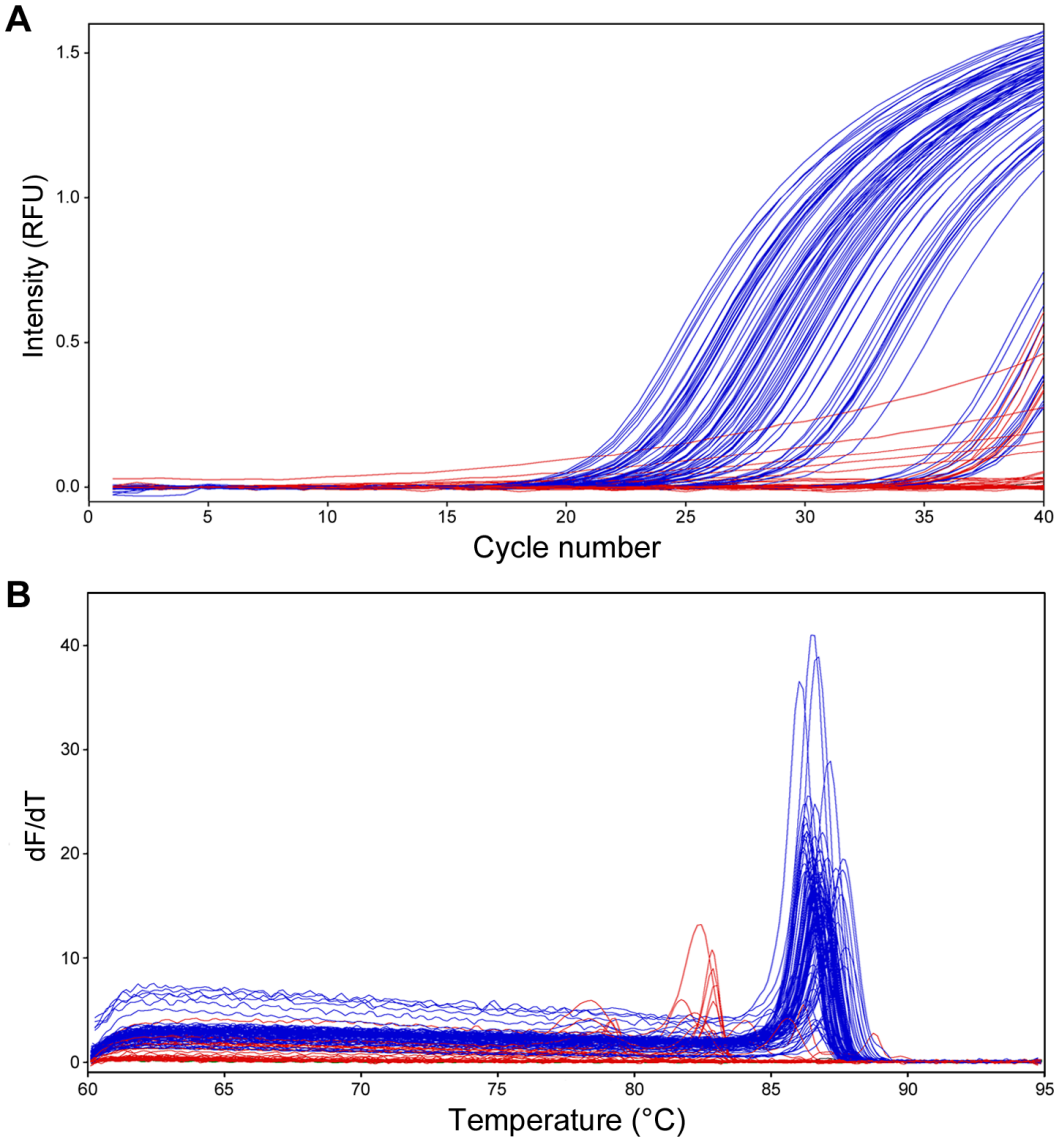
SYBR Green I assay for the detection of MNV in 158 mouse fecal samples. (A) Amplification curves and (B) melting curves. Blue lines show positive reactions and red lines show negative reactions. Green and black lines show FCV RNA and DVIM RNA, respectively, as negative controls. RFU, relative fluorescence units.

**Figure 5:**
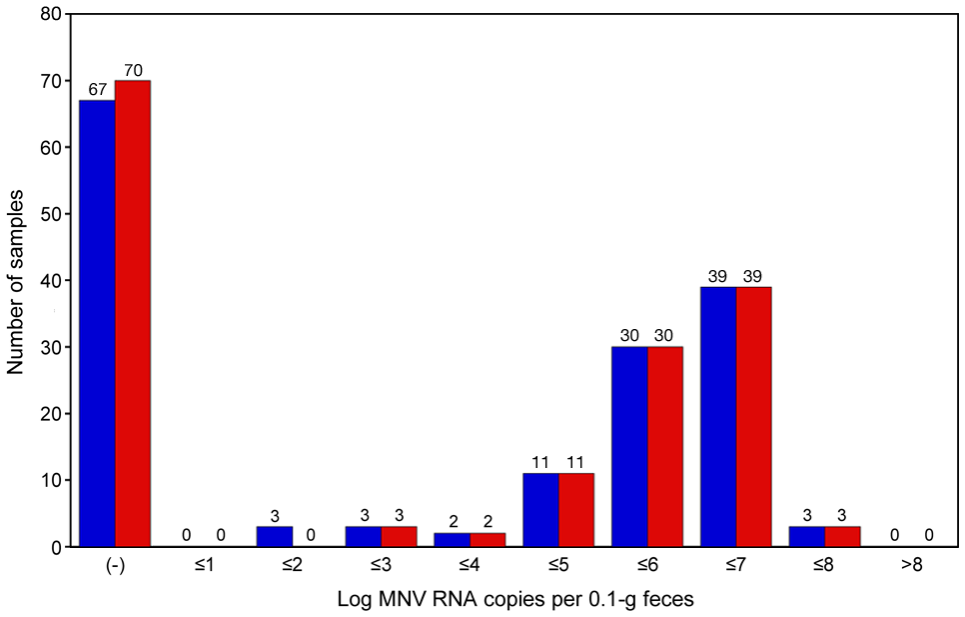
Diagnostic performance of SYBR Green I assay vs. TaqMan assay. MNV RNA genome copy number was measured by SYBR Green I assay and calculated from the 1.CW1 plasmid standard curve. SYBR Green I assay, blue bar; TaqMan assay, red bar.

To evaluate the performance of the SYBR Green I assay for the detection of MNV in clinical samples, 158 RNA samples from mouse feces were analyzed. The mice used for the fecal sample collection did not exhibit any clinical signs such as diarrhea, ruffled fur, and hunched back. The MNV-positive status was judged by the combination of the amplification curve analysis and the melting curve analysis (Figure 4). A sample with a Ct value of ≤35 and a T_m_ value of >86°C was considered positive. A sample with a Ct value in the range of 36–40 and a T_m_ value of >86°C was considered equivocal, and the sample was retested. If the result was replicated, the sample was considered positive. When the major peak in melting curve analysis was ≤86°C or ≥88°C, the sample was considered negative. Finally, the usability and diagnostic performance of the SYBR Green I assay were compared with those of the TaqMan assay. The SYBR Green I assay could be completed within 1 h, whereas the TaqMan assay required at least 3 h. Of 158 samples, 91 were confirmed positive by the SYBR Green I assay. MNV RNA in the feces was estimated in the range of 31 to 1.6×10^7^ copies/0.1 g feces, with the greatest distribution in the range of 1×10^5^ to 1×10^7^ copies/0.1 g feces (Figure 5). In contrast, 88 samples were confirmed positive by the TaqMan assay. The amount of MNV RNA in the 3 samples that were confirmed positive by only the SYBR Green I assay was 31, 74, and 82 copies/0.1 g feces. In addition, the melting curve analysis showed the presence of genetic variations among MNV detected in 91 samples. All samples confirmed negative by the SYBR Green I assay were also judged negative by the TaqMan assay. When viral RNA isolated from FCV and DVIM culture supernatants were examined, gene amplification was not observed.

## Discussion

Real-time RT-PCR assays for detecting MNV have been previously reported [31], [32], [38]–[40], [41]. Most of them are based on costly TaqMan technology; however, they cannot detect all currently known MNV strains with equal sensitivity. Thus, a broadly reactive, simple, and cost-effective real-time RT-PCR assay for detecting MNV isolates is required for routine laboratory diagnosis. The one-step real-time RT-PCR assay using a DNA-binding dye is ideal because such dyes are inexpensive, and in comparison with the TaqMan assay, this method reduces the chances for pipetting errors and cross-contamination. The melting curve analysis after real-time PCR not only assures reaction accuracy but also detects genetic variations [40], [44]. We therefore developed a one-step SYBR Green I real-time RT-PCR assay for routine laboratory diagnosis of MNV infection.

It is important to design the primers that do not amplify non-target sequences because the SYBR Green I dye binds to any double-stranded DNA. Primer pairs that theoretically detect 101 MNV strains were designed manually, and the best primer pair was selected on the basis of the detection sensitivity using S7-PP3 RNA. The selected primer pair F2 and R2 satisfied the 7 rules for primer design recommended by Innis and Gelfand [45], except for the GC content (%) of F2. The assessment of the primer specificity was performed with the Primer-BLAST program, NCBI [46]. When the program was limited to the analysis of the product length of <500 bp, R2 alone was shown to have the potential to synthesize a 196-bp PCR product of *Mus musculus* potassium voltage-gated channel, subfamily Q, member 1 (Kcnq1), mRNA (NCBI Reference Sequence: NM_008434). R2 has no mismatch between the last 8 nucleotides at the 3′ end of the primer and Kcnq1 mRNA. However, the unexpected gene amplification was not observed when fecal RNA samples derived from MNV-free mouse strains, guaranteed by a provider, were used as templates (data not shown).

To evaluate the broad reactivity of the SYBR Green I assay, 7 of 101 MNV strains were selected on the basis of the country of isolation, phylogenetic analysis of the amplified regions by Clustal Omega (http://www.ebi.ac.uk/Tools/msa/clustalo/) [47], and GC content of the PCR product without primer regions. The assay showed reproducibility and quantitative capacity for the detection of the 7 MNV plasmids. The amplification efficiency was almost equal among the plasmids, and the estimated detection limit was at least 10 copies/reaction. The melting curve analysis also showed reproducible results for the 7 MNV plasmids and was in agreement with the predicted melting profiles of the respective PCR products. Similar results were obtained in assays with MNV RNA substituted for the plasmids. The SYBR Green I assay also showed a high linear correlation between virus titer and Ct value. Accordingly, the SYBR Green I assay has the potential to detect various MNV with sensitivity, reproducibility, and sufficient quantitative capacity. In contrast, when the TaqMan assay was performed with 1000 copies of the 7 MNV plasmids, threshold cycles for the detection of Apo960 were delayed by 6 Ct compared with those for the detection of other 6 MNV [43]. The forward primer has a mismatch at position −5 of the 3′ end to the Apo960 genome sequence. This single point mismatch may cause approximately 64-fold underestimation of the Apo960 genome as Korn et al. reported on the Cobas TaqMan PCR assay for the human immunodeficiency virus-1 RNA quantification [48]. Thus, the SYBR Green I assay is expected to be more precise than the TaqMan assay in the quantification of viral RNA obtained from numerous MNV strains.

In a practical study using 158 mouse fecal samples, 155 results (88 positives and 67 negatives) obtained by the SYBR Green I assay were in 100% agreement with the results obtained by the TaqMan assay. However, 3 samples that tested positive in the SYBR Green I assay were negative in the TaqMan assay. The diagnostic performance of SYBR Green I assay was superior to that of the TaqMan assay as the results were reproducible by respective assays. Of 158 mouse fecal samples, 56 MNV positive samples were obtained from mice deposited from 11 laboratories or institutes in Japan at the RIKEN BRC. PCR products showed a diversity of T_m_ values ranging from 86.2°C to 87.7°C. The depositors are located in geographically distinct areas and the melting curve analyses of 56 MNV positive samples demonstrated genetic diversity. These results indicate that MNV is highly prevalent in laboratory mouse colonies in Japan and possesses genetic diversity. This gives the SYBR Green I assay an advantage over the TaqMan assay as the genetic diversity of MNV can be presumed without additional DNA sequencing analysis of the PCR product.

In conclusion, the SYBR Green I assay developed in this study is a reliable tool for the rapid and sensitive diagnosis of MNV infection in laboratory mouse colonies. This assay could also be applicable for the quantification of MNV RNA even when MNV strains other than MNV-1 are used as substitutes for HuNoV studies.

## Supporting Information

Table S1 **Murine norovirus strains used for primer design.**
(XLS)
